# Looking back to travel light: progenitor genomes empower hybrid potato breeding

**DOI:** 10.1093/plcell/koag319

**Published:** 2026-10-24

**Authors:** Linhan Sun

**Affiliations:** Assistant Features Editor, The Plant Cell, American Society of Plant Biologists, United States; Department of Plant Pathology and Environmental Microbiology, The Pennsylvania State University, University Park, PA 16802, United States

“The longer you can look back, the farther you can look forward,” Winston Churchill once famously remarked. This quote rings especially true for breeding of potato (*Solanum tuberosum*). Unlike crops such as maize, in which breeders routinely cross elite inbred lines to exploit hybrid vigor, potato breeding still relies largely on highly heterozygous tetraploid plants propagated clonally via tubers (Li et al. 2013). This heterozygosity can mask deleterious variants, which are genetic changes at evolutionarily conserved sites predicted to reduce fitness (Wu et al. 2023). When developing homozygous diploid lines through repeated selfing, previously masked deleterious alleles can become homozygous and thus reduce vigor and yield. Thus, this heavy “genetic baggage” has become a major bottleneck for redesigning potato as a seed-propagated diploid crop for hybrid breeding. Although recent breeding has successfully purged many large-effect deleterious variants, existing inbred lines still carry numerous minor-effect variants, which collectively reduce their fitness and can compromise the performance of resulting hybrids (Zhang et al. 2021). Looking back at the domestication history of potato may reveal how the frequencies of these variants changed over time and help breeders “look forward” to the future of hybrid potato breeding.

In new work in *The Plant Cell*, **Weizhi Liu, Yong Hu, Zihui Ding, and colleagues (**Liu et al. 2026**)** have taken another major step in this direction. The authors expanded an existing panel of the wild potato progenitor *Solanum candolleanum* (CND) from 119 to 221 accessions by sequencing 102 additional materials, broadening both the geographic coverage and the genetic diversity of this panel (Fig. 1a). The CND panel was compared with 279 landraces, which represent farmer-selected potato varieties adapted to local environments (Fig. 1a). The average genetic distance between CND accessions and landraces was significantly greater than that among landraces. This greater divergence suggests that CND accessions could be more promising than landraces as sources of genetic variation for enhancing hybrid vigor.

**Figure 1:**
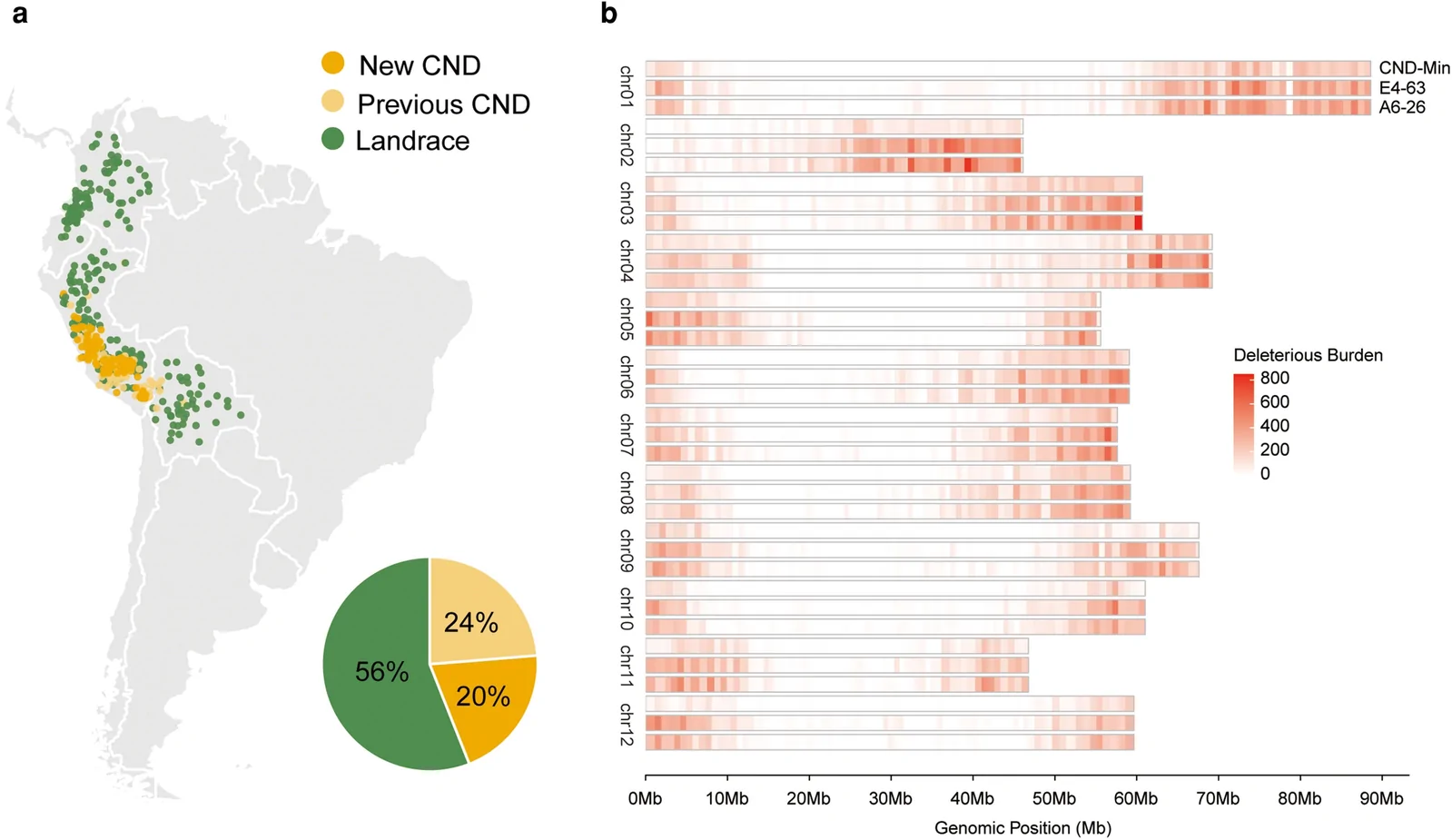
a**)** Geographic distribution of the CND and landrace accessions analyzed in this work. b**)** Predicted deleterious burden of a progenitor-assisted genome built from low-burden CND segments (CND-min), compared with 2 potato inbred lines (E4-63 and A6-26). Adapted from Liu et al. (2026), Figs. 1A and 5B.

The authors first mapped the landscape of deleterious variants in the expanded CND panel. CND accessions had a lower frequency of deleterious variants than landraces. Here, common variants refer to variants with an allele frequency over 0.05 in the sampled population, whereas variants found in 5% or fewer were classified as rare. On average, individual CND accessions also carried fewer common deleterious variants than landraces. Because such common variants are widespread among landraces, they would be difficult to purge if using landraces alone. The CND panel therefore could serve as a valuable source of alternative, lower-burden genetic variations.

Next, the authors asked how domestication reshaped the landscape of deleterious variants. They focused on selective sweep regions, where strong selection during domestication is thought to substantially reduce local genetic diversity. They identified these genomic regions by looking for regions with lower nucleotide diversity in landraces than in CND. Intriguingly, deleterious variants were disproportionately concentrated in these regions. These regions are also characterized by strong genetic linkage and reduced recombination. Consequently, when favorable and deleterious alleles are closely linked, selection for a favorable allele can carry the nearby deleterious alleles along, making the latter harder to remove. Of the deleterious variants whose frequencies differed significantly between CND and landraces, about 65% were more frequent in landraces. Among the deleterious variants with significantly higher allele frequencies in landraces than in CND, about 20% shifted from being minor alleles in CND to major alleles in landraces, and 227 even reached near-fixation (a frequency of at least 90%) in landraces. Notably, these frequency-shifted variants, particularly those near fixation, were highly enriched in selective sweep regions, making them extremely difficult to purge using landraces alone. CND accessions may come to rescue by providing alternative alleles that became rare or were even lost during domestication.

Finally, to test whether progenitor genomes might help reduce the cumulative burden of minor-effect deleterious variants remaining in current inbred lines, the authors examined deleterious variants carried by 2 inbreds, A6-26 and E4-63 (Zhang et al. 2021), and compared the frequencies of these variants in CND and landraces. These variants occurred at lower frequencies in CND than in landraces, again highlighting CND as a rich source of alternative alleles. Consistent with this idea, a progenitor-assisted genome built from low-burden CND segments was predicted to carry 56% lower deleterious burden than the 2 inbred genomes (Fig. 1b). Its predicted burden was also comparable to that of tomato, a vigorous inbred crop closely related to potato. Thus, progenitor-assisted genome design could theoretically bring the deleterious burden of potato inbreds closer to that of an established inbred crop, a feat that might not be possible using landraces alone.

Overall, this work provides a new roadmap for shedding the deleterious burden accumulated during potato domestication: rather than focusing on introgression of individual favorable genes, breeders could use low-burden genome segments from progenitors for genome-wide optimization of inbred lines. Experimental validation will still be needed to evaluate the practical value of this strategy, but the genomic resources generated in this work provide a strong foundation for such efforts. This strategy may also benefit other clonally propagated crops suffering from similar issues of heavy deleterious burden accumulated during domestication, such as cassava (Ramu et al. 2017). Guided by ancestors, future breeding programs may be able to travel more lightly, leaving much of the genetic baggage behind.

## Recent related articles in *The Plant Cell*

- Fan and Whitaker (2024) reconstructed the recent domestication of cultivated strawberry using whole-genome data and clarified how wild progenitors contributed to modern cultivars.
- Jiang et al. (2024) examined the genetic load contributed by transposable elements (TEs) in Arabidopsis and linked increased TE load during range expansion to smaller effective population size, increased transposition, and selective sweeps.
- Zhao et al. (2026) used naturally occurring loss-of-function (LoF) variants in Arabidopsis to distinguish deleterious gene losses from potentially adaptive ones. They also developed a genome-wide LoF burden test that asssoicate gene loss with molecular phenotypes.

## Data Availability

No new data was generated for this article.
